# RNA-Seq Reveals an Integrated Immune Response in Nucleated Erythrocytes

**DOI:** 10.1371/journal.pone.0026998

**Published:** 2011-10-27

**Authors:** Davinia Morera, Nerea Roher, Laia Ribas, Joan Carles Balasch, Carmen Doñate, Agnes Callol, Sebastian Boltaña, Steven Roberts, Giles Goetz, Frederick W. Goetz, Simon A. MacKenzie

**Affiliations:** 1 Institute of Biotechnology and Biomedicine, Universitat Autònoma de Barcelona, Barcelona, Spain; 2 Unitat de Fisiologia Animal, Departament de Biologia Cel.lular, Fisiologia i Immunologia, Facultat de Ciències, Universitat Autònoma de Barcelona, Bellaterra (Barcelona), Spain; 3 School of Aquatic and Fishery Sciences, University of Washington, Seattle, Washington, United States of America; 4 School of Freshwater Sciences, University of Wisconsin-Milwaukee, Milwaukee, Wisconsin, United States of America; University of Texas, United States of America

## Abstract

**Background:**

Throughout the primary literature and within textbooks, the erythrocyte has been tacitly accepted to have maintained a unique physiological role; namely gas transport and exchange. In non-mammalian vertebrates, nucleated erythrocytes are present in circulation throughout the life cycle and a fragmented series of observations in mammals support a potential role in non-respiratory biological processes. We hypothesised that nucleated erythrocytes could actively participate via ligand-induced transcriptional re-programming in the immune response.

**Methodology/Principal Findings:**

Nucleated erythrocytes from both fish and birds express and regulate specific pattern recognition receptor (PRR) mRNAs and, thus, are capable of specific pathogen associated molecular pattern (PAMP) detection that is central to the innate immune response. *In vitro* challenge with diverse PAMPs led to *de novo* specific mRNA synthesis of both receptors and response factors including interferon-alpha (IFNα) that exhibit a stimulus-specific polysomal shift supporting active translation. RNA-Seq analysis of the PAMP (Poly (I∶C), polyinosinic∶polycytidylic acid)-erythrocyte response uncovered diverse cohorts of differentially expressed mRNA transcripts related to multiple physiological systems including the endocrine, reproductive and immune. Moreover, erythrocyte-derived conditioned mediums induced a type-1 interferon response in macrophages thus supporting an integrative role for the erythrocytes in the immune response.

**Conclusions/Significance:**

We demonstrate that nucleated erythrocytes in non-mammalian vertebrates spanning significant phylogenetic distance participate in the immune response. RNA-Seq studies highlight a mRNA repertoire that suggests a previously unrecognized integrative role for the erythrocytes in other physiological systems.

## Introduction

The function of the vertebrate erythrocyte is agreed to be oxygen-transport by respiratory globin pigments. Across non-mammalian vertebrates, nucleated erythrocytes are present in the circulation often with extended longevity throughout the life cycle of the organism. Intriguingly, the potential contribution of nucleated erythrocytes as transcriptionally-active cells to non-respiratory physiological processes has not been systematically addressed in non-mammalian species. Instead, red blood cell (EC) functions in non-mammalian vertebrates have tacitly been assumed to follow a highly conserved role as observed in mammalian anucleated erythrocytes.

The immune response is understood to have a modular structure mainly formed by sub-sets of activated leukocytes responding to different combinations of PAMPs via PRR-mediated recognition [1]. These cellular interactions are modulated by extrinsic local regulation by soluble factors including cytokines that form complex networks of cellular communication. Immune-specific mRNAs including PRRs and cytokines have been shown to exhibit considerable promiscuity for expression throughout the diverse cellular phenotypes involved in an immune response [2]. This in turn suggests that a nucleated cell of hematopoietic origin in circulation should have the potential to respond and contribute to the immune response given its ability to move freely throughout the body.

The origins and definitive descriptions of the evolution of the erythrocyte lineage in vertebrates is lacking. The ontogeny of vertebrate erythropoiesis has been well characterised in mammals, birds, amphibians and bony fish [3]–[5]. In mammals, nucleated erythrocytes (mnEC) produce regulatory factors such as cytokines in response to changes in the bone marrow micro-environment [6]–[7]. We hypothesised that the presence of a nucleus and transcriptional/translation machinery could confer to non-mammalian erythrocytes an active, ligand-induced transcriptional re-programming leading to a functional role that contributes to the immune response. Our results show that trout and chicken erythrocytes are capable of eliciting PAMP-specific responses that correspond to an active cellular response that likely regulates leukocyte activity. The observed modulatory role of erythrocytes in non-mammalian vertebrates, the presence of many transcripts that interact with other physiological systems, and the vast number of these cells in the circulation leads us to suggest that a reorganisation of the current thinking of integrated immunity/physiology in non-mammalian systems may be required.

## Results

### Nucleated erythrocytes contain the cellular machinery to respond to PAMPs

**Figure 1:**
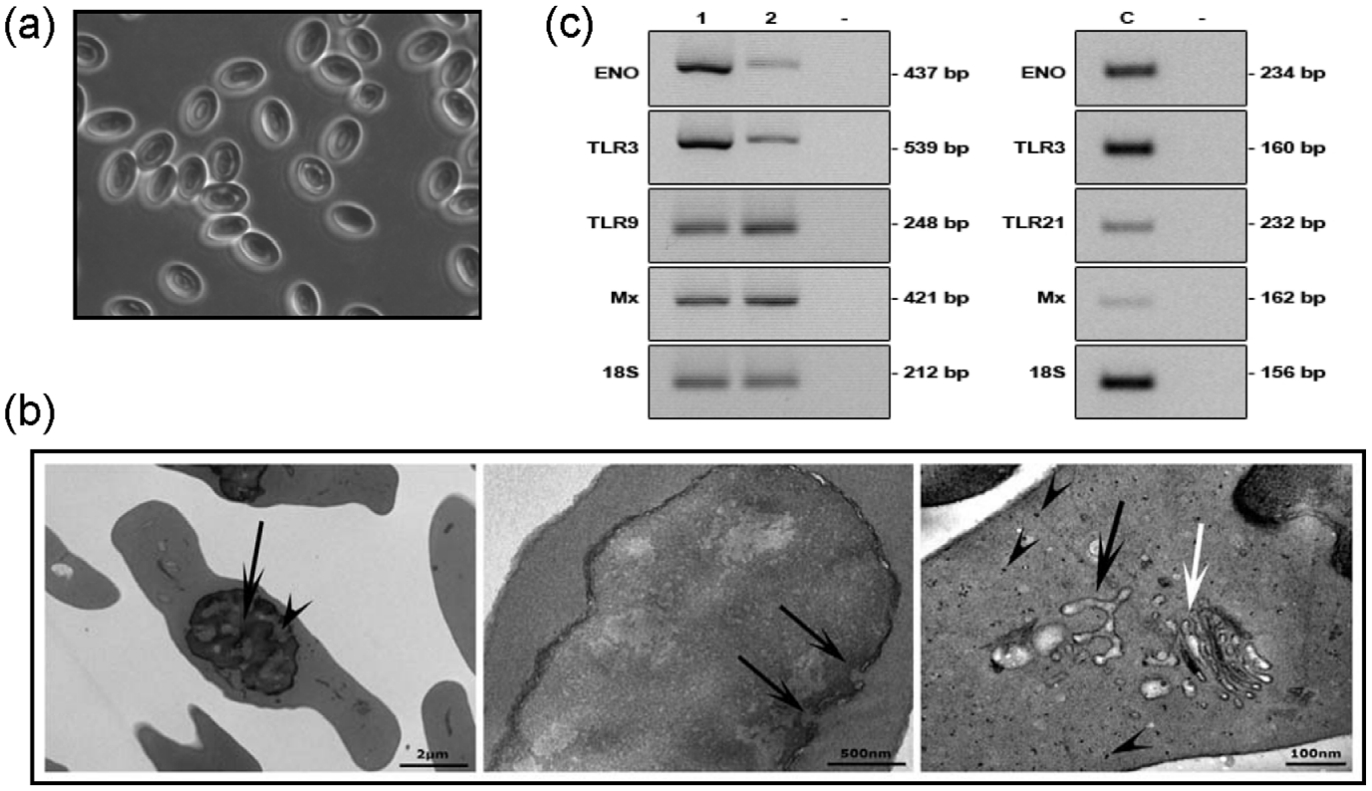
Model description of rainbow trout erythrocytes. (**a**) General micrograph of cultured rainbow trout erythrocytes (×50). (**b**) TEM of trout erythrocyte cultures. *Left panel*, nucleus show condensed (arrow) and decondensed (arrowhead) chromatin; *middle panel*, trout erythrocyte nucleus with nuclear pores (arrows); *right panel*, erythrocyte with ribosomes (arrowheads), smooth endoplasmic reticulum (black arrow) and Golgi apparatus (white arrow). (**c**) Specific mRNA transcript expression (RT-PCR) in trout and chicken erythrocytes (ENO, enolase; TLR 3, 9 and 21, Toll-like receptors; Mx, myxovirus resistance 1). *Left panel*, 1) density gradient isolated cultured trout erythrocytes under control conditions; 2) cell sorted trout erythrocyte population from density gradients. *Right panel*, c) specific mRNA transcript expression in density gradient isolated cultured chicken erythrocytes under control conditions. One representative of four individuals is shown.

Rainbow trout erythrocytes (tECs) are present in the circulation at a concentration of approximately 1×10^9^ cells/ml and show a typical oval morphology (**Fig. 1a**) as in almost all non-mammalian vertebrates with few exceptions [8]–[9]. TEM analysis highlighted major cellular features throughout the erythrocyte population including nuclear pores, de-condensed chromatin, ribosomes, golgi bodies and endoplasmic reticulum [10] (**Fig. 1b**). Consistent identification of large tracts of intracellular organs in erythrocytes varied considerably between individuals possibly due to the erythrocyte maturation status in the circulation [11]. Trout erythrocytes were purified by density gradient centrifugation and cell-sorted to ascertain erythrocyte cell culture homogeneity (>99.9% pure, **Fig. S1**). Initial RT-PCR of candidate immune system related genes in erythrocytes from both trout and chicken (cEC) showed that erythrocytes contain a range of different mRNAs (**Fig. 1c** **, Fig. S2**). Thus nucleated vertebrate ECs possess an active transcriptional (de-condensed chromatin) cellular morphology and the cellular machinery for the production of proteins and contain mRNAs coding for reception, integration and response to external stimuli.

### Regulation of PAMP responsive mRNAs

**Figure 2:**
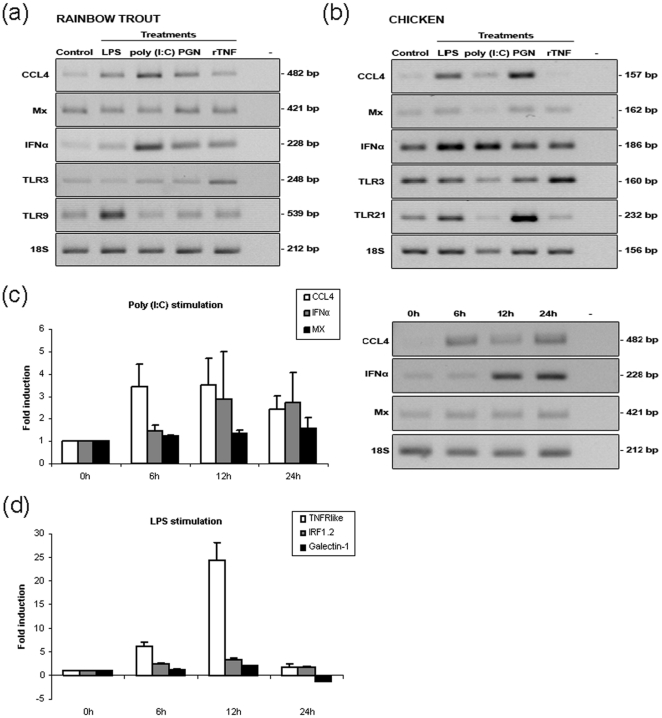
Analysis of specific mRNA transcript expression in cultured trout and chicken erythrocytes after PAMP or cytokine stimulation. (**a**) Response of trout and (**b**) chicken erythrocytes after 12 hours exposure to: 50 µg/ml of LPS, 50 µg/ml of poly (I∶C), 5 µg/ml of PGN and 50 ng/ml of rTNF. CCL4, Mx, IFNα, TLR3 and TLR9 (TLR21 in chicken) mRNA abundance was analyzed by RT-PCR and 1.2% agarose gel electrophoresis. One representative of 3 and 4 individuals is shown for trout and chicken respectively. (**c**) RT-PCR analysis of the tEC response over time (6–24 h) to 50 µg/ml of poly (I∶C), densitometry data shown as fold changes (mean ± std.dev., n = 4 individuals) with respect to 18S rRNA. (**d**) Absolute Q-PCR analysis of the tEC response over time control (6–24 h) to 50 µg/ml of LPS, fold changes (mean ± std.dev., n = 4 individuals) in respect to specific transcript copy number.

The presence of several PRR mRNAs in both trout (Toll-like receptor (TLR) 3 and 9) and chicken (TLR3 and 21) erythrocytes (**Fig. 1c** **, Fig. S2**) suggest that ECs are able to detect and specifically respond to different PAMPs. PAMPs have been shown to trigger the activation of an immune response in all metazoans thus far studied [1]. To explore the triggering of a specific response in trout and chicken erythrocytes we first tested 3 different PAMPs (bacterial lipopolysaccharide (LPS), peptidoglycan (PGN) and poly (I∶C)) and a recombinant cytokine, rainbow trout tumor necrosis factor-alpha (rTNF). Due to poly (I∶C)-mediated interference in quantitative-PCR (Q-PCR) analyses, all data for poly (I∶C) experiments are shown as semi-quantitative RT-PCR and densitometry. *De novo* synthesis of specific chemokine (CCL4) and IFNα mRNA transcripts after PAMP and rTNF treatments was observed in both species and TLR mRNA responses varied between individuals (**Fig. 2a–b** **, Fig. S3**). Increases in specific transcript synthesis was both time and PAMP-dependent peaking from 6–24 hours for CCL4 and IFNα transcripts and tumor necrosis factor receptor-like (TNFR-like), interferon regulatory factor 1.2 (IRF1.2) and Galectin-1 (decreasing at 24 hours) transcripts under poly (I∶C) and LPS stimulation respectively (**Fig. 2c–d**).

**Figure 3:**
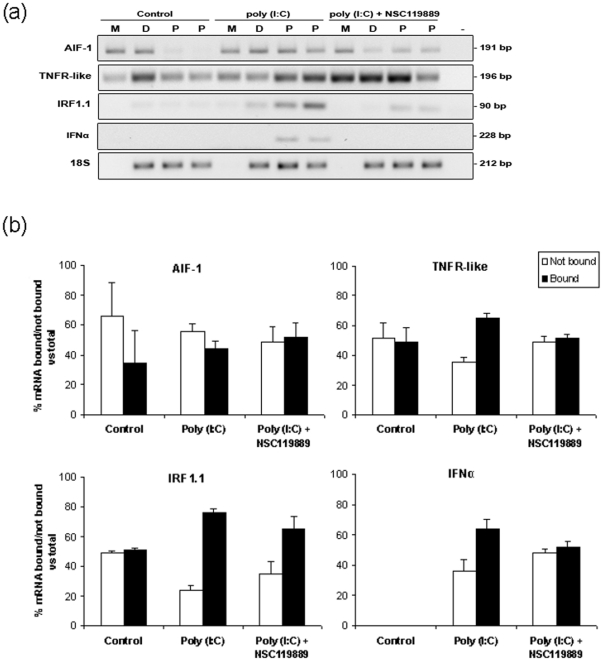
Analysis of polysome-bound mRNA regulation in cultured trout erythrocytes after poly (I∶C) stimulation. (**a**) RT-PCR analysis of polysome-bound mRNAs (AIF-1, TNFR-like, IRF1.1 and IFNα) under control and after poly (I∶C) stimulation. M, ribosome free mRNA; D, mono and disome bound mRNA; and P, polysome bound mRNA obtained after polysome gradient centrifugation. NSC119889 (200 µM) was added to inhibit polysome formation. (**b**) Densitometry analysis (n = 3) of % bound or non-polysome specific mRNA in respect to the total specific mRNA abundance.

As relative mRNA abundance measurements from total cellular RNA samples do not reflect post-transcriptional processing we performed polysomal gradient analyses since the association of polyA mRNAs with polysomes is a strong indicator of translation and has been shown to be an important regulatory mechanism in the immune response [12]–[13]. A typical vertebrate polysome profile was obtained from tECs (**Fig. S4**) and a poly (I∶C)-dependent polysomal shift was observed for allograft inflammatory factor-1 (AIF-1), TNFR-like and IRF1.1 mRNA transcripts (**Fig. 3**). The protein synthesis initiation inhibitor, NSC119889, inhibited mRNA-polysome formation showing that these mRNAs depend upon a 5′-mediated/cap-dependent initiation of protein synthesis. Therefore specific mRNAs are induced and differentially regulated by PAMP-PRR interactions leading to specific transcriptional and post-transcriptional responses in ECs.

### Erythrocytes activate PAMP-specific re-modeling of the transcriptome

**Figure 4:**
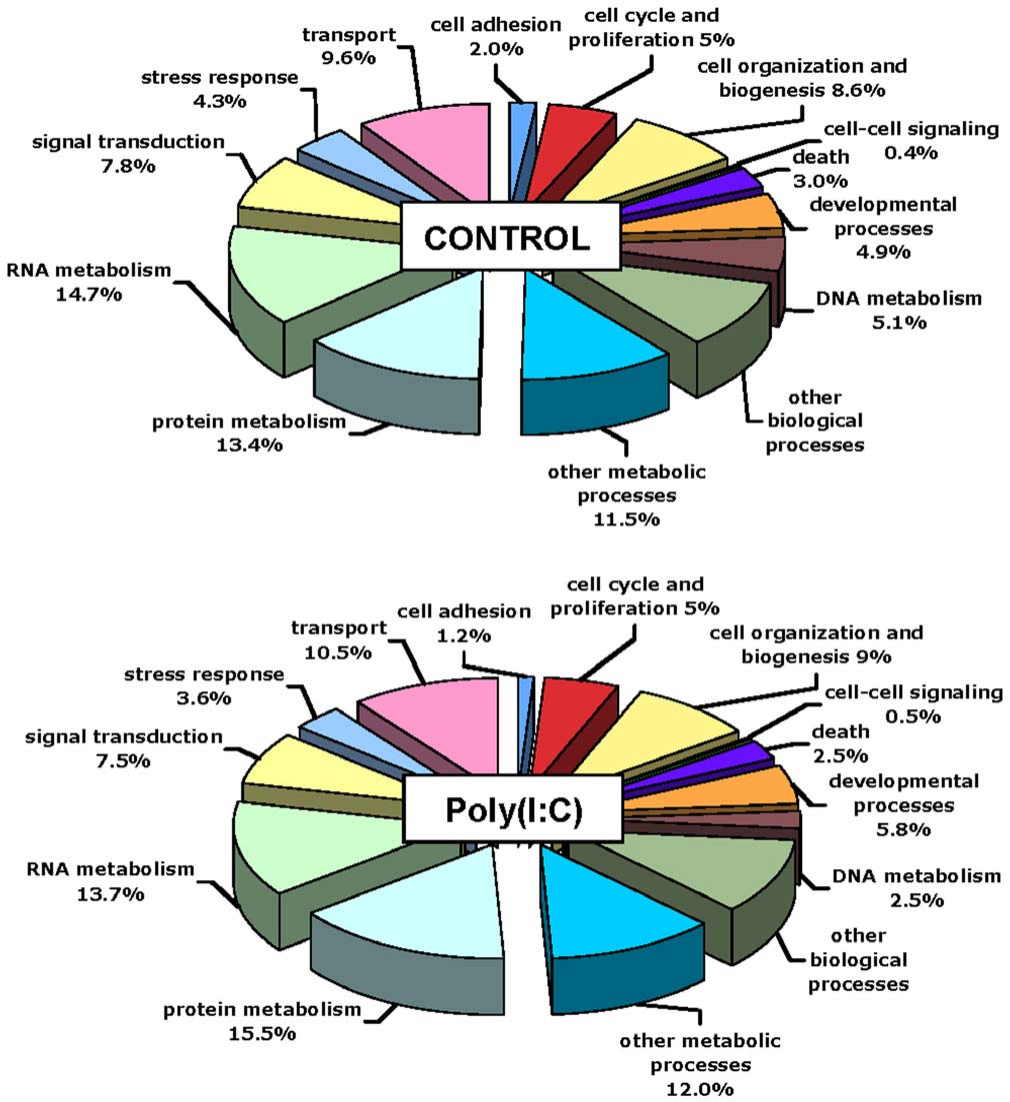
Gene Ontology representation of tEC stimulated with or without poly (I∶C). Pie charts of the percent of transcripts within functional categories for genes regulated >2 fold in the control versus poly (I∶C) libraries. Genes regulated >2 fold were divided into functional categories using CateGOrizer (http://www.animalgenome.org/bioinfo/tools/countgo/).

To explore the transcriptomic response of the tEC, we constructed 2 pooled libraries (control and 12 h poly (I∶C) stimulated, n = 8) for RNA-Seq analysis using the ABI SOLiD 3 platform. After quality trimming, a total of 80×10^6^ reads (average 44 bp/read), were obtained in approximately a 1∶1 ratio for each library. No sequenced rainbow trout genome is available therefore RNA-Seq analysis and gene annotations were carried out by mapping the sequences against the SIGENAE contig database for rainbow trout (http://www.sigenae.org/). RNA-Seq analysis resulted in the expression from both libraries of 25940 features (corresponding to contigs). A direct comparison of both libraries (unique contig number in each pool) revealed 2378 unique features that were up-regulated and 1475 unique features were down-regulated by 2-fold or higher in the poly (I∶C) treated library compared to the control library (**Table S1**). In general, poly (I∶C) appeared to increase the percentage of transcripts coding for proteins involved in DNA metabolism and stress and decreased transcripts for proteins involved in protein metabolism and developmental processes (**Fig. 4** **)**. Even though RNA-Seq analysis revealed thousands of genes to be differentially regulated by 2 fold or greater between libraries, the expression of only 55 genes were calculated by DESeq analysis to be statistically different between control and poly (I∶C) stimulated tEC at p≤0.1 (**Table S2**). This number dropped to 41 genes at p≤0.05. However, for sequencing we used duplicate pools of RNA from the incubations of tEC obtained from 8 individual tEC cultures. While DESeq analysis can theoretically be performed on the data from nonreplicated samples, when used in this fashion it will be very conservative and will indicate that only a fraction of the differentially regulated genes are statistically different [14]. This strongly indicates that sequencing should be replicated if statistical differences with RNAseq data are being analyzed. Thus a qualitative comparison of gene ontology categories (GOSlim) represented by transcripts regulated (FC>2) in RNA-Seq highlights changes in key biological processes such as DNA metabolism and cellular stress response however replication will be required to accurately describe over-represented GO categories (**Fig. S4b**).

Within all of the transcripts found by RNA-Seq to be regulated 2 fold or greater between the two libraries (**Table S1**), the erythrosome contained transcripts for hormone receptors including those for estrogen (P) (P = higher in poly (I∶C) library than control; C = higher in control than poly (I∶C)), androgen (P), prostaglandin E (C), leukotriene B4 (C), vitamin D (P), insulin-like growth factor (C), and luteinizing hormone (P). Pivotal enzymes involved in both eicosanoid and steroid synthesis and metabolism such as steroidogenic acute regulatory protein (C), aromatase (C), and cyclooxygenases I & II (C) were observed. Transcripts for growth factors and other modulatory proteins including myostatin (P), transforming growth factor B (P), activin B (and receptor) (P), angiopoietin (C), and angiotensinogen (P) were observed as well as cytokines including type I interferon (P), interleukin 16 (C), TNF superfamily members 13 (P) and 14 (C), CCL4 (P), various cytokine and chemokine receptors and potential pathogen recognition receptors including toll receptors (P) II, 5, 9, 13 and 20, and scavenger receptors (P) (**Table S1**). Thus trout erythrocytes appear to regulate large cohorts of mRNAs in response to poly (I∶C) however a more robust analysis with increased biological replicates will be necessary to quantify the exact magnitude and intensity of this response.

### Erythrocytes modulate the anti-viral macrophage response

**Figure 5:**
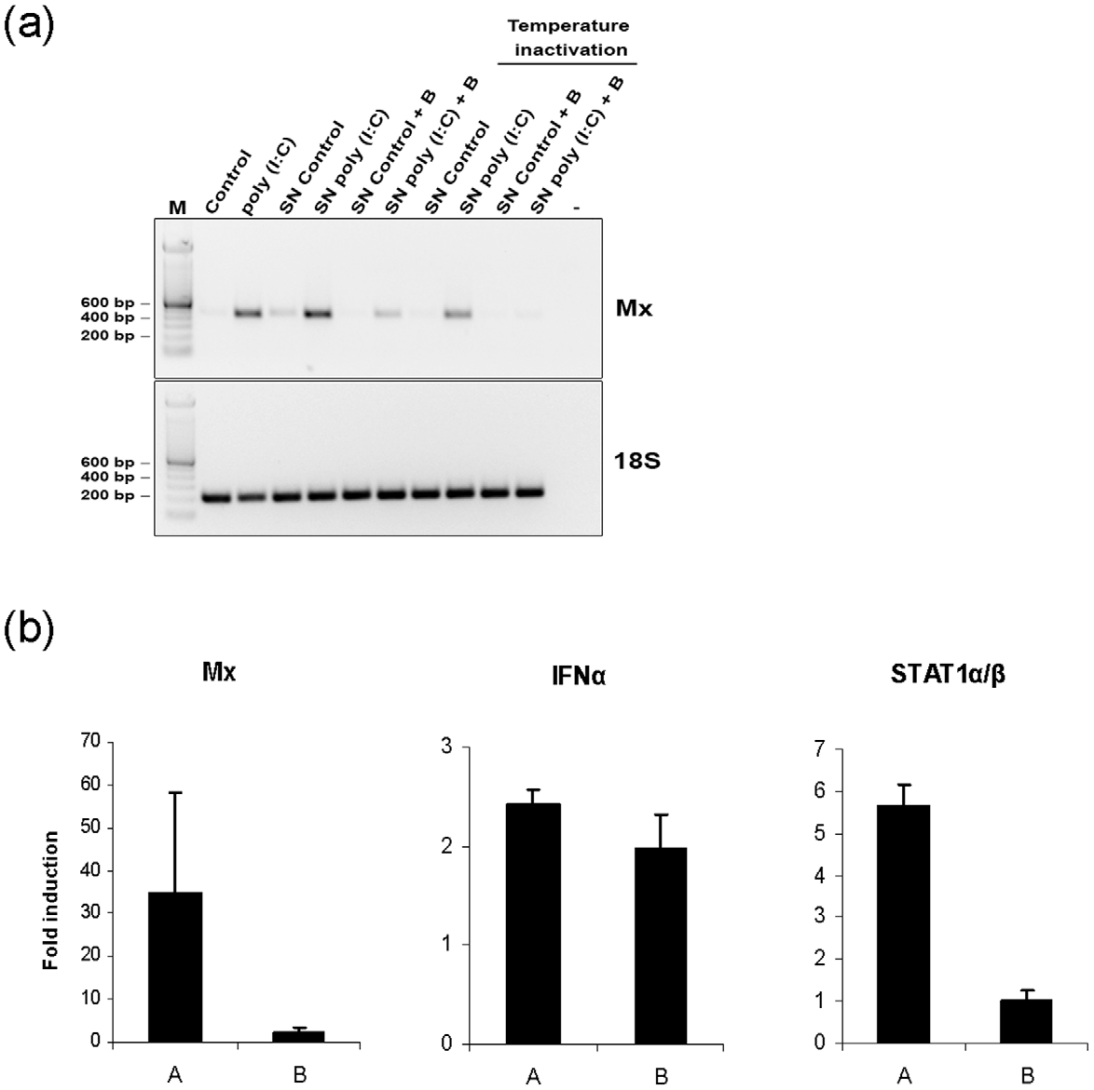
Effects of conditioned medium from poly (I∶C) stimulated tECs upon the anti-viral response in adherent trout monocyte/macrophages. (**a**) Rainbow trout macrophage Mx mRNA abundance analyzed by RT-PCR after 12 h incubation with tEC supernatants. eCM were incubated with benzonase to remove poly (I∶C) (50 µg/ml) and/or incubated at 95°C for 10 min. (**b**) Mx, IFNα and STAT1α/β mRNAs abundance after 12 h incubation with tEC supernatants. Data shown as fold change (mean ± std.dev, n = 3). A, poly (I∶C) stimulated erythrocyte supernatants benzonase-treated vs control supernatant benzonase-treated; B, poly (I∶C) stimulated erythrocyte supernatants benzonase- and temperature-treated vs control supernatant benzonase- and temperature-treated.

Multi-directional cellular communication is a key function that directs the intensity, scale and development of an immune response by remodelling the transcriptomic landscape. RNA-Seq analysis revealed a number of mRNAs that code for regulatory soluble factors induced in tECs in response to poly (I∶C) treatment. To examine a possible functional role for modulating the leukocyte response, the effect of conditioned medium derived from poly (I∶C)-activated tECs (eCM-24 h incubation) was assayed with differentiated trout macrophages. Mx (myxovirus resistance 1), STAT1α/β (signal transducer and activator of transcription 1 alpha/beta) and IFNα mRNAs were chosen as markers for the anti-viral response and were up-regulated in macrophages stimulated with eCM supernatants derived from poly (I∶C)-stimulated tECs treated with benzonase to remove excess poly (I∶C) (**Fig. S4b**). This effect could be blocked with heat treatment of the conditioned mediums (**Fig. 5a–b**). Therefore, activation with a double-stranded RNA mimic, a specific TLR3 ligand in fish, causes tECs to secrete bioactive, temperature-labile molecules that modulate the macrophage anti-viral response indicating a functional role for the erythrocyte in the anti-viral immune response.

## Discussion

The primary function of nucleated erythrocytes in non-mammalian vertebrates has been tacitly accepted to be O_2_ and CO_2_ transport, and has never been questioned in a systematic manner. Here we demonstrate that nucleated erythrocytes from rainbow trout and chicken develop specific PAMP-PRR responses at the level of the transcriptome and, at least in trout, erythrocytes stimulated with poly (I∶C) secrete thermo labile molecules that can modulate the anti-viral response in macrophages. Our studies indicate that nucleated erythrocytes from two vertebrate groups spanning significant evolutionary time possess the cellular and molecular machinery to specifically respond to pathogens and likely contribute to the regulation of an immune response. Therefore, this ability may well extend to all non-mammalian vertebrates.

We suggest that the current paradigm of the leukocyte-driven immune response that is mainly derived from mammals may require modification for the vast majority of vertebrate species that have nucleated erythrocytes. The current paradigm for organisation of the immune response suggests that the cells of the immune system are organised into PRR-driven networks and multi-cellular effector modules that act in a coordinated fashion to eliminate invading pathogens from the organism [1]. Our data, far from challenging the above shows that nucleated erythrocytes are capable of PRR-driven responses, produce soluble factors that modulate leukocyte activity and therefore play an active role in the non-mammalian immune response. Evidence in support of this can be gathered from a limited set of observations derived from a diverse group of organisms, including mammals, where functional responses in erythrocytes include: haemoglobin-derived anti-microbial responses [15], glycophorin A-mediated pathogen sink [16], endothelial nitric oxide synthase (eNOS)-like protein and activity [17], specific human immunodeficiency virus (HIV)-1 binding [18], IFNα production [19], hormone binding [20] and cortisol receptor 1 (CR1)-dependent immune complex clearance [21]. Thus in the erythrocytes a set of biological processes relevant to immunity and the immune response are present. Furthermore the dual functionality of haemoglobin, active in the anti-microbial response [15] and in respiratory gas exchange in both invertebrates and vertebrates, provides an interesting evolutionary backdrop for the evolution of the erythrocytes in vertebrates.

The global transcriptome perspective obtained from RNA-Seq analysis also points toward a more complex and integrated role for erythrocytes in regulatory physiology. The presence of mRNAs relevant to specific receptor-response networks from diverse physiological systems including endocrine, immune and reproductive uncovers the exciting possibility that ligand regulated transcription in the abundant erythrocytes may have a fundamental and previously unrecognized regulatory function in homeostatic balance. Extrapolating from mRNA abundance measurements (absolute transcript number) erythrocytes do not express exceptionally high levels of mRNA in comparison to PAMP-activated leukocytes, however given their abundance in the circulation, several millions of mRNA transcripts/ml of blood could be expected. Furthermore, transcripts produced in response to PAMP stimulation were polysome-bound suggesting a rapid biological response for both cellular and secreted proteins. Considering the transcript diversity uncovered by RNA-Seq, one is tempted to speculate that circulating erythrocytes may constitute a regulatory tissue interface between physiological compartments in the organism with largely unknown properties.

In conclusion, here we demonstrate that nucleated erythrocytes in non-mammalian vertebrates likely participate in an active fashion in the immune response and contain an mRNA repertoire integrating functions from other physiological systems.

## Methods

### Animals

Adult rainbow trout (*Oncorhynchus mykiss*) of approx. 150 g mean weight were obtained from J. Antrés fish farm (St. Privat, Girona). Fish were transported to aquarium facilities at the Universitat Autònoma de Barcelona and held in stock tanks at 15°C under a standard photoperiod of 12 h light/12 h dark for two weeks before experimentation. Trout were fed at 0.5% body weight per day. Experimental protocols for blood sampling, sacrifice and organ isolation have been reviewed and approved by the Ethics and Animal Welfare Committee of the Universitat Autònoma de Barcelona, Spain (AGL2009-10677). Animals were sacrificed with a lethal concentration of ethyl 3-aminobenzoate methanesulfonate (MS-222, 0.2 g/l, Sigma, France) for head kidney dissection or anesthetized with clove oil (40 ppm) for blood sampling.

### Erythrocyte cell culture

Trout blood was obtained from the caudal vein and chicken blood, obtained from the Experimental Unit del Prat (IRTA, Spain), from the heart using heparinized syringes. Trout (tEC) and chicken (cEC) erythrocytes were obtained by consecutive density gradient centrifugations (720×g, Ficoll 1.007; Lymphoprep, Reactiva, Spain) and washed twice in 1× PBS. tECs were resuspended in DMEM (PAA, Germany) supplemented with 10% heat-inactivated FBS (PAA, Germany) and 100 µg/ml Primocin (Invivogen, France) at a density of 7.5×10^6^ erythrocytes/ml in six well cell culture plates and cultured at 18°C (tEC) or 37°C (cEC) and 5% CO_2_. Cells were cultured O/N before experimentation. tEC were analyzed using a MoFlo cell sorter (Dako Cytomation, USA). Excitation settings used for the flow cytometer were laser 488 nm, 150 mW. Forward Scatter (FSC) was collected with a PMT tube in a linear scale and side scatter (SSC) was collected at 90 degrees in a logarithmic scale. Windows for sorting were defined on FSC, SSC and pulse width in order to exclude aggregates. For stimulations, lipopolysaccharide from *Escherichia coli* 026:B6 (LPS, 50 µg/ml), peptidoglycan from *E. coli* 0111:B4 (PGN, 5 µg/ml), poly (I∶C) (50 µg/ml) and recombinant tumor necrosis factor alpha from rainbow trout (rTNF, 50 ng/ml) were added and trout and chicken cultures were incubated for the 24 h. To produce conditioned mediums (eCM), tECs were stimulated for 24 h with 50 µg/ml poly (I∶C) and culture supernatants collected. Supernatants containing poly (I∶C) were treated with or without ultrapure Benzonase (Sigma-Aldrich, France) (500 U/ml tEC supernatant, 18°C O/N) and temperature inactivated at 95°C for 10 min.

### Monocyte/macrophage cell culture

Adherent trout monocyte/macrophages were isolated as previously described [22]. Before stimulation, differentiated macrophages were incubated in serum free medium for 3 h. Cells were incubated either with poly (I∶C) (10 µg/ml) as a positive control or eCMs for 12 hours.

### Electron microscopy

tEC were centrifuged at 400×g, 5 min and pellets fixed for 2 h at 4°C with 2.5% glutaraldehyde, 2% paraformaldehyde in a sodium phosphate buffer (0.1 M, pH 7.4). Pellets were subsequently washed with phosphate buffer. Samples were then incubated with 1% osmium tetraoxide in sodium phosphate buffer (0.1 M, pH 7.4) for 2 h at 4°C. Sequential acetone washes (50% to 100%) were used to dehydrate samples and finally, the samples were fixed in an Epon resin and visualized with Jeol Jem-2011 transmission electron microscopy (Jeol LTD., Japan).

### Gene expression studies

Total RNA was extracted from tEC and cEC cultures (45×10^6^ cells) following manufacturer's instructions with minor modifications (TriReagent, Sigma, France). RNA (400 ng) was used to synthesize cDNA with SuperScript III Transcriptase (Invitrogen, Spain) and oligo-dT primer (Promega, Spain). Conventional RT-PCR was carried out to analyze gene expression and polysome gradient associated mRNAs. 1 µl of cDNA was used as a template for reactions with specific primers (**Table S3**) and Amplitaq DNA polymerase (Biotools). As a control, ribosomal 18S was amplified from the same cDNA samples. Products were separated on agarose gels, stained with GelGreen Nucleic Acid Gel Stain (Biotium, USA) and visualized with AlphaImager 2200. Q-PCR was used to analyze gene expression in eCM stimulated macrophages. Q-PCR was carried out with SYBR Green I (Bio-Rad, Spain) using a 1∶50 dilution of cDNA, 500 nM of primers and 20 µl final volume. The ribosomal 18S was used to normalize gene expression using a 1∶500 dilution and quantification was done according to Pfaffl method corrected for efficiency for each primer set [23]. Absolute Q-PCR was carried out under the same conditions using a 10^9^ to 10^2^ copies/µl dilution of plasmid DNA (pGEM, Promega, USA). Standard curves (Ct-Threshold cycle versus log copy number) were constructed for sample copy number determination. All Q-PCR was performed using a MyiQ instrument (BioRad, Spain).

### Polysome gradients

tECs were stimulated for 24 h with either 50 µg/ml poly (I∶C) or 200 µM NSC119889 plus poly (I∶C). Polysomes were obtained as previously described [12]–[13] with minor modifications. Briefly, 1.8×10^8^ cells were lysed in NP-40 lysis buffer (0.2% NP-40, 40 mM KCl, 3 mM MgCl_2_, 5% Glycerol, 10 mM Tris-HCl, 5 mM Dithiothreitol, 50 Units RnaseOUT (Life Technologies S.A., Spain)) and cytoplasmic extracts loaded onto pre-prepared 15–40% sucrose gradients. Gradients were centrifuged at 140,000×g at 4°C for 2 h and 30 minutes (Beckmann, SW55Ti rotor). Total RNA was purified from resulting fractions (400 µl/fraction) with Tri-Reagent (Sigma-Aldrich) following the manufacturer's guidelines with minor modifications. From the 8 fractions obtained we pooled to provide 4 final fractions. Fraction M, ribosome free mRNA; D, mono and disome bound mRNA; and P, polysome bound mRNA.

### RNA-Seq

tEC cultures (n = 8) were stimulated with poly (I∶C) 50 µg/ml for 12 h. Samples were prepared with the SOLiD Whole Transcriptome Analysis Kit (Applied Biosystems) according to the manufacturer's protocol. Briefly, mRNA was fragmented using RNase III and fragmentation was verified with an Agilent Bioanalyzer 2100 using the RNA 6000 Pico Chip Kit. Samples were hybridised (Adaptor Mix A), ligated, and reverse transcribed. cDNA was subjected to electrophoresis on a Novex 6% TBE-Urea Gel (Invitrogen, USA), stained with SYBR Gold (Invitrogen, USA), and the region corresponding to 100–200 nucleotides was excised from the gel. In-gel PCR was performed and reactions purified with the PureLink PCR Micro Kit (Invitrogen, USA) according to the manufacturer's protocol and analysed with an Agilent Bioanalyzer 2100 using the DNA 1000 Chip Kit (Agilent). Sequencing was perform using the SOLiD3 System (Applied Biosystems) according to the manufacturer's protocol. A total of four panels were sequenced corresponding to two panes for each library. All sequence analysis was performed with CLC Genomics Workbench (CLC Bio) software. Initially, sequences were trimmed based on quality scores of 0.05 [24]–[25] and the number of ambiguous nucleotides (>2 on ends). Sequences smaller than 30 bp were also removed. RNA-Seq analysis was performed using sequence assembly pom7 of *Oncorhynchus mykiss* from SIGENAE (http://www.sigenae.org/). All contig sequences and corresponding annotations were downloaded from the Trout EST contig browser (http://public-contigbrowser.sigenae.org:9090/Oncorhynchus_mykiss/index.html). Expression values were measured as RPKM (reads per kilobase of exon model per million mapped reads) [26] with an unspecific match limit of 5 and maximum number of mismatches of 2 (CLC Genomics Workbench; CLC Bio). RNA-Seq count data from the poly (I∶C) and control groups were analyzed by DESeq [14] to assess statistically different gene expression. Settings for nonreplicated data were used in the analysis.

## Supporting Information

Figure S1 Cell sorting of cultured trout erythrocytes using MoFlo cell sorter (Dako Cytomation). Cells were first gated using pulse width and forward scatter (FSC) (left). R1 population was gated by FSC and side scatter (SSC) (right) in order to exclude aggregates.
(PDF)

Figure S2 (**a**) Agarose gel electrophoresis showing the PCR products of different mRNAs in purified rainbow trout erythrocytes under control conditions. ENO, enolase; CR, glucocorticoid receptor; PU.1 (spleen focus forming virus (SFFV) proviral integration oncogene spi1); TNF, tumor necrosis factor. Ribosomal 18S was used as a loading control. M, molecular weight marker. (**b**) Pathogen recognition receptor (PRR) expression in control purified rainbow trout erythrocytes. Abundance of TLR3, TLR9 and PGRP mRNAs is shown on the left panel. Right panel is the negative controls (-). M, molecular weight marker.
(PDF)

Figure S3 Semi-quantification of RT-PCR products (densitometry) from tEC and cEC stimulated over 24 h with different PAMPs. Density of the bands was normalized with 18S and fold change calculated over the control. (trout n = 3, chicken n = 4)
(PDF)

Figure S4 (**a**) Electrophoresis (virtual total RNA; Bioanalyzer 2100, Agilent Technologies) of cytoplasmic mRNA fractionated in a 15–40% sucrose gradient (Polysome-bound mRNAs). Lane 1–8 represents fractions relative to density sedimentation; lane 9, total RNA from rainbow trout macrophages; and M, molecular weight marker. (**b**) Benzonase (500Units/ml) digestion of 50 µg/ml of poly (I∶C) in cell culture medium (DMEM, 10%FBS).
(PDF)

Table S1 All genes that were regulated at 12 h 2 fold or greater in tEC control and poly (I∶C) libraries determined by RNA-seq analysis. ABI SOLiD sequences were mapped against the rainbow trout SIGENAE contigs (http://public-contigbrowser.sigenae.org:9090/Oncorhynchus_mykiss/index.html) and the RPKM expression values were determined. Table provides number of mapped reads/contig, expression levels, difference and fold change in RPKM between control and poly (I∶C) libraries. SIGENAE contigs were aligned against the Gene Ontology Database (version GO.200801) and the top blast hit (accession number and description) is provided for each contig.
(XLS)

Table S2 Results of DESeq analysis on the RNAseq data for poly (I∶C) and control tEC. For “Fold Change,” values of ≥1 indicate contigs for which expression in poly (I∶C)-stimulated tECs were greater than controls, while values of <1 indicate higher expression in control vs poly (I∶C)-stimulated tECs. Annotation of SIGENAE contigs (http://www.sigenae.org/) was performed using a local, custom “Best Blast” program that aligns sequences first by BLASTX against the NCBI nonredundant (nr) protein database, then depending on the BLASTX results, against the NCBI nucleotide (nt) database by BLASTN.
(XLS)

Table S3 Rainbow trout and chicken specific primers for PCR.
(JPG)
