# Supplementary figures and images for: RNA-Seq Reveals an Integrated Immune Response in Nucleated Erythrocytes
Source: PLoS One. 2011 Oct 27;6(10):e26998. doi: 10.1371/journal.pone.0026998 (PMC3203173; doi:10.1371/journal.pone.0026998)

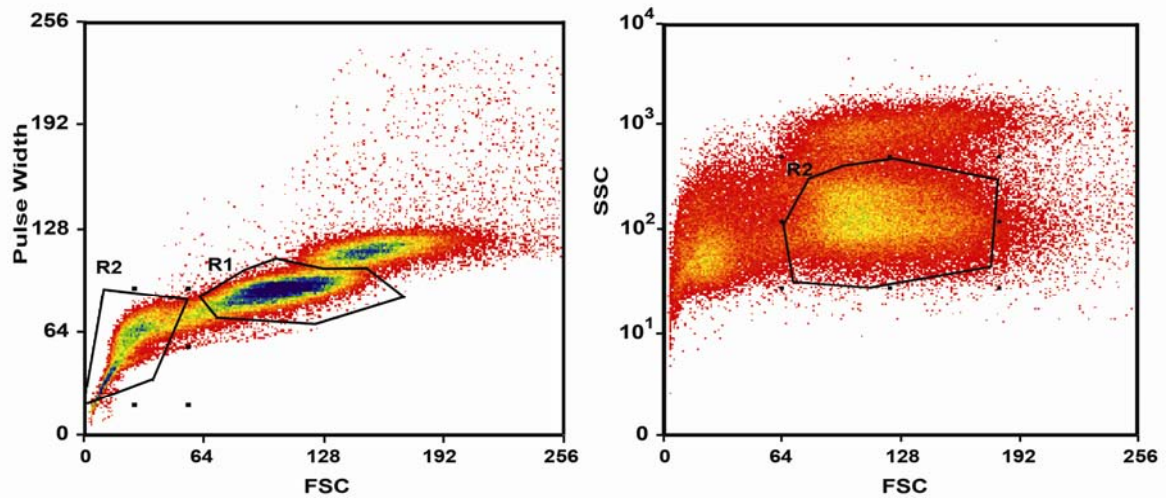

Supplement: Figure S1 — Cell sorting of cultured trout erythrocytes using MoFlo cell sorter (Dako Cytomation). Cells were first gated using pulse width and forward scatter (FSC) (left). R1 population was gated by FSC and side scatter (SSC) (right) in order to exclude aggregates. (PDF) [file pone.0026998.s001.pdf]

**a**

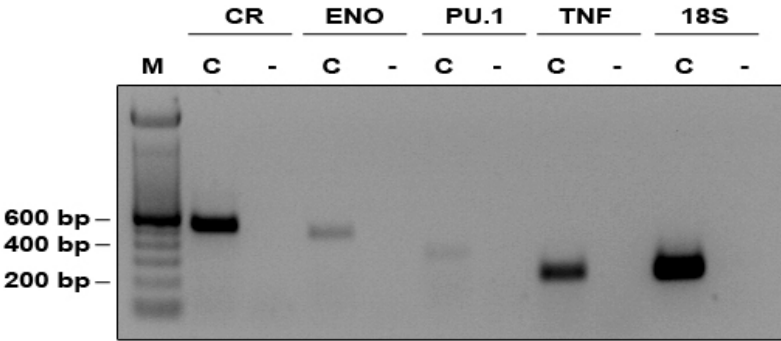

**b**

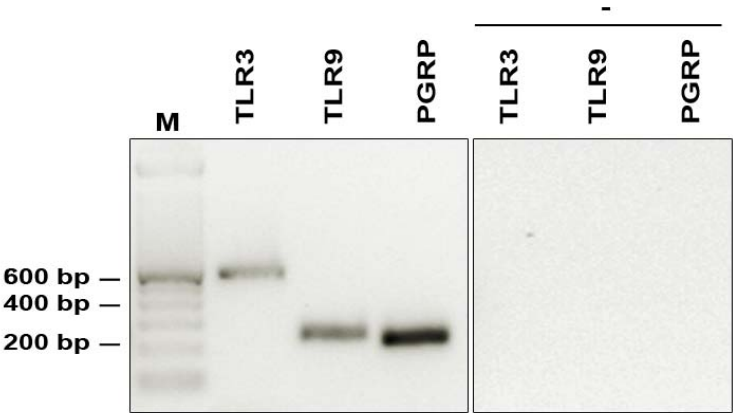

Supplement: Figure S2 — (a) Agarose gel electrophoresis showing the PCR products of different mRNAs in purified rainbow trout erythrocytes under control conditions. ENO, enolase; CR, glucocorticoid receptor; PU.1 (spleen focus forming virus (SFFV) proviral integration oncogene spi1); TNF, tumor necrosis factor. Ribosomal 18S was used as a loading control. M, molecular weight marker. (b) Pathogen recognition receptor (PRR) expression in control purified rainbow trout erythrocytes. Abundance of TLR3, TLR9 and PGRP mRNAs is shown on the left panel. Right panel is the negative controls (-). M, molecular weight marker. (PDF) [file pone.0026998.s002.pdf]

## Rainbow trout densitometry

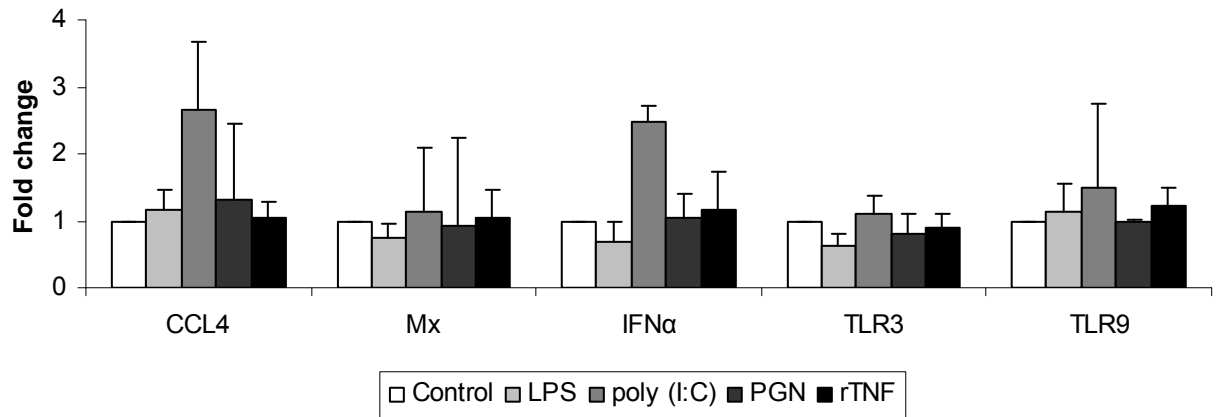

## Chicken densitometry

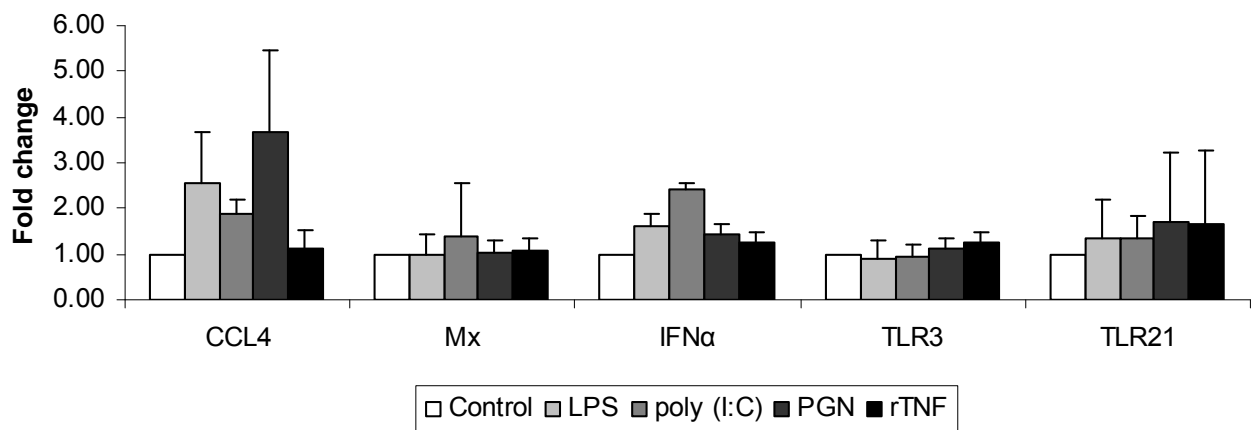

Supplement: Figure S3 — Semi-quantification of RT-PCR products (densitometry) from tEC and cEC stimulated over 24 h with different PAMPs. Density of the bands was normalized with 18S and fold change calculated over the control. (trout n = 3, chicken n = 4) (PDF) [file pone.0026998.s003.pdf]

**a**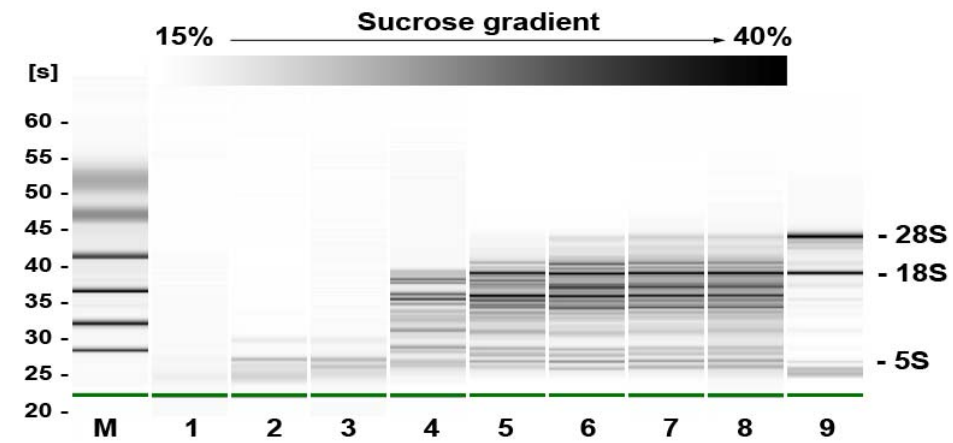**b**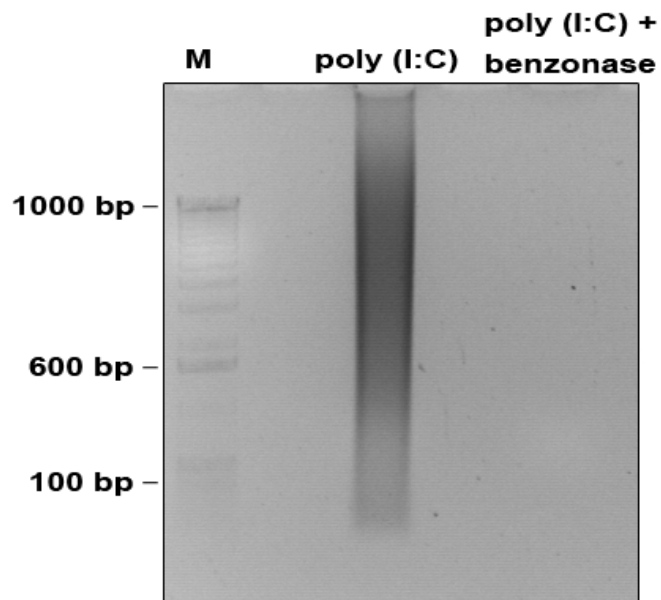

Supplement: Figure S4 — (a) Electrophoresis (virtual total RNA; Bioanalyzer 2100, Agilent Technologies) of cytoplasmic mRNA fractionated in a 15–40% sucrose gradient (Polysome-bound mRNAs). Lane 1–8 represents fractions relative to density sedimentation; lane 9, total RNA from rainbow trout macrophages; and M, molecular weight marker. (b) Benzonase (500Units/ml) digestion of 50 µg/ml of poly (I∶C) in cell culture medium (DMEM, 10%FBS). (PDF) [file pone.0026998.s004.pdf]

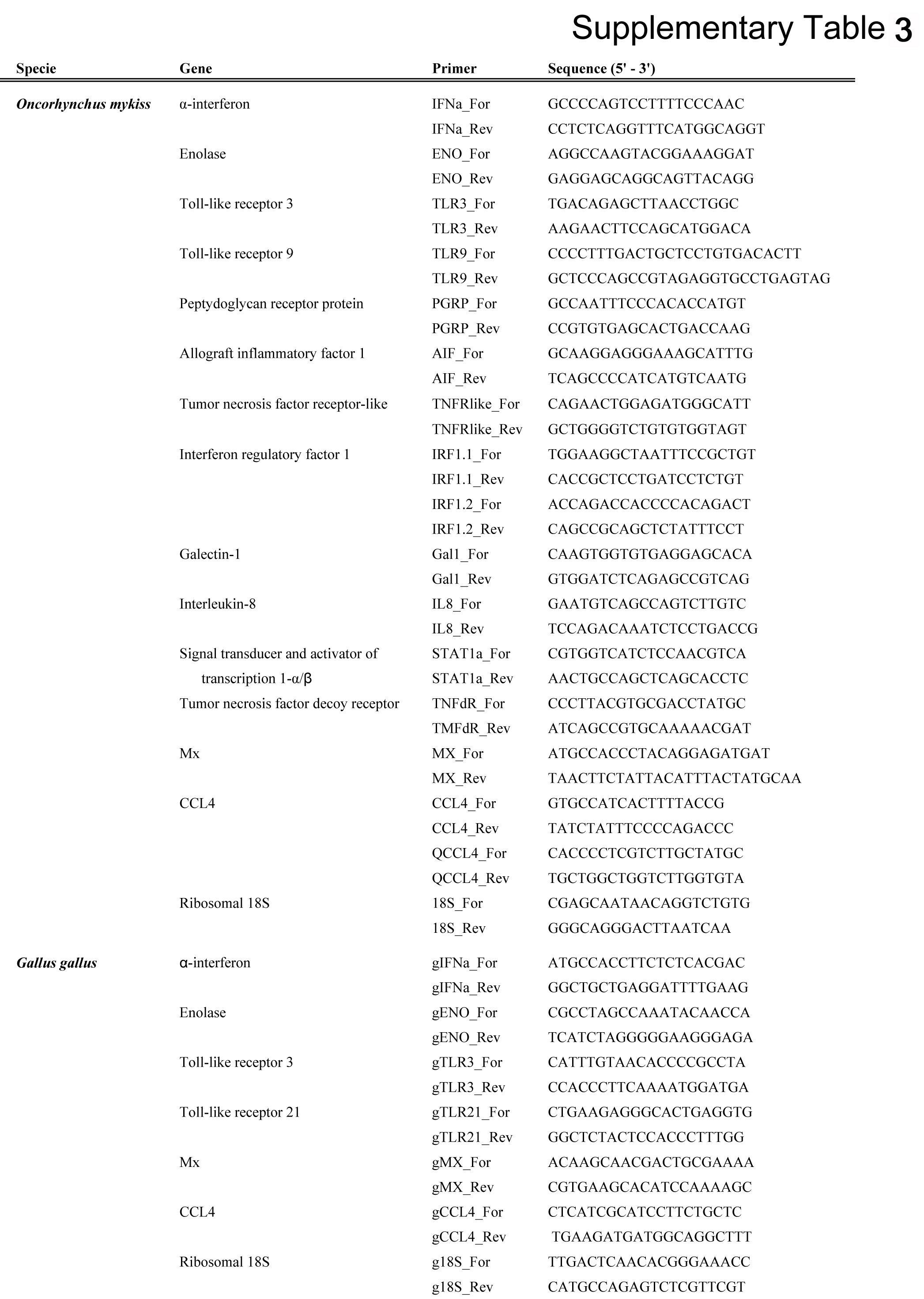

Supplement: Table S3 — Rainbow trout and chicken specific primers for PCR. (JPG) [file pone.0026998.s007.jpg]
